# Impact of Culinary Procedures on Nutritional and Technological Properties of Reduced-Fat Longanizas Formulated with Chia (*Salvia hispanica* L.) or Oat (*Avena sativa* L.) Emulsion Gel

**DOI:** 10.3390/foods9121847

**Published:** 2020-12-11

**Authors:** Tatiana Pintado, Claudia Ruiz-Capillas, Francisco Jiménez-Colmenero, Ana M. Herrero

**Affiliations:** Institute of Food Science, Technology and Nutrition (ICTAN-CSIC), José Antonio Novais 10, 28040 Madrid, Spain; tatianap@ictan.csic.es (T.P.); claudia@ictan.csic.es (C.R.-C.); fjimenez@ictan.csic.es (F.J.-C.)

**Keywords:** fresh sausages, longanizas, grilling, chia and oat EGs, nutritional and health claims, technological properties

## Abstract

This paper evaluates how grilling, a traditional culinary procedure for fresh meat products, affects the composition and technological properties of healthy longanizas formulated with chia (*Salvia hispanica* L.) (C-RF) and oat (*Avena sativa* L.) (O-RF) emulsion gels (EGs) as animal fat replacers. The use of EGs, regardless of whether they contain chia or oat, improved longaniza performance during cooking as they lost less (*p* < 0.05) water and fat. The composition of cooked sausages was affected by their formulation, particularly those with chia EG (C-RF) which featured the highest polyunsaturated fatty acid content, mainly due to the higher level of α-linolenic fatty acid (1.09 g/100 g of product). Chia and oat EGs in C-RF and O-RF allow longanizas to be labeled with nutritional and health claims under European law. In general, this culinary procedure increases (*p* < 0.05) the lightness, lipid oxidation and texture parameters of all samples.

## 1. Introduction

Plant-based ingredients are used to enhance health-promoting bioactive components in meat products [1,2]. Oat bran (*Avena sativa* L.) is an example that has been widely used in the preparation of a number of meat products, mainly providing them with soluble fiber (β-glucans), minerals (Mg, Fe, Cu, etc.), vitamins and phenolic compounds [3,4,5]. Chia (*Salvia hispanica* L.) is a plant-based ingredient that is gaining in popularity due to its interesting nutritional properties deriving from higher α-linolenic acid levels, insoluble dietary content and minerals (Ca, Fe, Mg, Mn, etc.) and vitamins [6]. Hence, chia has been added to different meat products to provide them with various bioactive compounds [7,8,9,10,11,12,13] and attractive technological properties (water and fat binding capacity, emulsifying and gelling properties, etc.) [14,15]. Due to their emulsifying and gelification potential, both chia and oat have been used in oil structuring processes to obtain new lipid materials which, thanks to their characteristics, can be used as animal fat substitutes. With this aim in mind, it is worth noting the use of EGs to reformulate fresh and cooked meat products due to their technological properties (texture, color, etc.) and their capacity to deliver bioactive compounds [16,17]. Especially in certain fresh meat products such as longanizas, chia and oat EGs have been deemed as suitable animal fat replacers and vehicles of bioactive compounds, allowing them to make different nutritional and health claims [18,19] and also feature optimal sensorial, technological and microbiological properties [20]. However, marketing these fresh sausages as healthier alternatives based on their composition may not be entirely accurate given the variety of processes that must be carried out prior to consumption (storage, thawing, cooking, etc.) [21]. Longanizas, for example, like other fresh meat products, need to be cooked prior to consumption. Cooking methods (roasting, grilling, frying, etc.) and conditions (time, temperature, heating rate, etc.) have a noticeable impact on the balance of (healthy/unhealthy) compounds and energy value [21]. Furthermore, lipid oxidation or other technological variations occur as a consequence of high temperatures [21,22].

It therefore makes sense that in the case of fresh meat products designed and produced to be healthier, we need information on their real nutritional and technological properties after having been subjected to different cooking procedures. Hence, the first aim was to produce a healthier fresh meat product (longanizas) by adding chia or oat EGs as animal fat replacers and delivery systems of certain bioactive compounds to evaluate the impact of grilling (selected because it is one of the most common ways to cook this type of product) on the composition and technological properties of longanizas. The second aim of the study was to determine how nutritional and health claims were affected by this cooking process. Longanizas elaborated with only animal fat (pork back-fat) (normal and reduced fat content) were used as reference.

## 2. Materials and Methods

### 2.1. Oil-in-Water Emulsion Gels and Longanizas Preparation

Oil-in-water (O/W) emulsion gels (EGs) and longanizas (fresh sausages) were prepared as reported by Pintado et al. [20]. Briefly, two different EGs, one containing chia (*Salvia hispanica* L.) flour and the other oat (*Avena sativa* L.) bran, were used as animal fat replacers in the preparation of longanizas. These EGs were formulated with 20% olive oil, 58% water, 2% gelling agent based on alginate (0.73% sodium alginate, 0.73% CaSO_4_ and 0.54% sodium pyrophosphate) and 20% chia flour or oat bran depending on the desired formulation [16,17].

Four different longanizas were prepared with the same quantity of pork meat (60 g/100 g product). Two were formulated with only animal fat (pork back-fat) and used as the reference (all-animal fat): one with normal fat content (29 g pork back-fat/100 g product) called P-NF and the other with reduced fat content (7.25 g pork back-fat/100 g product) called P-RF. Additionally, two different reduced-fat longanizas were formulated replacing 90% of pork back-fat with 27 g/100 g of chia or oat EGs, with the final products denominated C-RF and O-RF, respectively. All the samples contained 4% of commercial seasoning for fresh sausages.

### 2.2. Cooking Method for the Longanizas and Weight Loss

Longanizas were cooked on an electric grill. Four samples were used for each formulation. This cooking method was chosen because it is the one that is most used for this kind of product. Preliminary cooking trials were performed to establish the cooking conditions required to achieve a meat core temperature of 70 °C. Sausages were weighed and cooked for 2 min at 210 ± 4 °C on an electric grill (Princess classic multigrill type 2321, Tilburg, The Netherlands). All sausages were then cooled at room temperature (20–22 °C) for 30 min, dabbed with a paper towel to remove visible exudate and then weighed to calculate weight loss determined by weight difference (four determinations). Results were expressed as a percentage of the initial weight. Samples were stored under chilled conditions (3 ± 2 °C) until analysis.

### 2.3. Composition and Energy Content of Longanizas

#### 2.3.1. Proximate Composition

Moisture and ash contents were determined in triplicate according to official methods [23]. Fat content was evaluated in triplicate following Bligh and Dyer [24]. Protein was measured in quadruplicate with a Nitrogen Determinator LECO FP-2000 (Leco Corporation, St Joseph, MI, USA). All determinations were performed on both raw and grilled samples.

#### 2.3.2. Fatty Acid Profile

Fatty acid content was determined (in triplicate for each type of sample) for both raw and grilled samples by saponification and bimethylation using C13:0 (7015-U Supelco PUFA No.2 Animal Source; Sigma-Aldrich Co., St. Louis, MO, USA) as the internal patron. Fatty acid methyl ester (FAME) was analyzed as described in Pintado et al. [20]. Fatty acids were expressed as g of fatty acid/100 g of product.

#### 2.3.3. Energy Content

Energy content was calculated based on 9 kcal/g for fat and 4 kcal/g for protein and carbohydrate [25].

### 2.4. Technological Properties

#### 2.4.1. Color and pH Determination

Color (CIE-LAB tristimulus values, lightness, L*; redness, a*; and yellowness, b*) was determined for raw and grilled sausage cross-sections using a Konica Minolta CM-3500 D Chroma Meter (Konica Minolta Business Technologies, Tokyo, Japan). Ten determinations were carried out for each sample type.

pH values were determined in quadruplicate for each formulation of longaniza using an 827 Metrohm pH-meter (MetrohmAG, Herisau, Switzerland) at room temperature on homogenates (1:10 *w*/*v* sample/distilled water ratio) of raw and cooked samples.

#### 2.4.2. Texture Analysis

Kramer shear force (KSF) was performed using a miniature Kramer (HDP/MK05) cell with a 5-bladed head to perform a shearing test. Kramer shear tests were carried out at room temperature on sections of 2 cm previously weighed. A 25 kg load cell was used. Force was exerted to a compression distance of 25 mm at 0.8 mm/s crosshead speed using a TA-XT.plus Texture Analyzer (Texture Technologies Corp. Scarsdale, NY, USA). KSF values were calculated as the maximum force per g of sample (N/g). Five measurements were taken on both raw and grilled sausages for each formulation.

#### 2.4.3. Lipid Oxidation Stability

Lipid oxidation was evaluated as a function of change in thiobarbituric acid-reactive substances (TBARs) [8]. TBAR determinations for each sample and formulation (raw and cooked) were performed in triplicate.

### 2.5. Statistical Analysis

One-way analysis of variance (ANOVA) was performed to evaluate statistical significance (*p* < 0.05) attributable to sample formulations (expressed in figures and tables with different superscript letters a, b, c, etc.) and two-way ANOVA was performed to evaluate statistical significance (*p* < 0.05) attributable to formulations and the effect of cooking (expressed in tables with different superscript numbers 1, 2) using the SPSS program (v.24, IBM SPSS Inc.; Chicago, IL, USA). Formulation and cooking were assigned as fixed effects and replication as a random effect. Results are given in terms of mean values and standard error of the mean. Least square differences (LSD) were used to compare mean values and formulations, while Tukey’s HSD test was used to identify significant differences (*p* < 0.05) between formulations and cooking procedures.

## 3. Results and Discussion

### 3.1. Cooking Method for the Longanizas and Weight Loss

**Figure 1:**
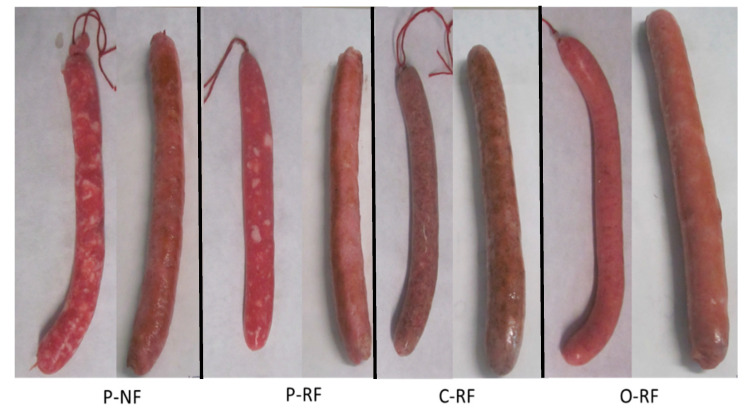
Appearance of longanizas: raw (left) and grilled cooked (right) (for each type of sample). Longanizas formulated with normal (P-NF) and reduced (P-RF) fat content (both with all-animal fat), and reduced-fat content sausages replacing 90% of pork back-fat with chia (C-RF) or oat (O-RF) emulsion gels (EGs).

Fresh meat products such as longanizas must be cooked and the electric grill is commonly used. Figure 1 shows the different appearances of raw and grilled samples.

This culinary procedure involves weight loss which furnishes information on the ability of the products to retain water and fat during processing that could alter the composition of sausages. Weight loss in longanizas cooked on an electric grill ranging from 6.71% to 24.73% was affected (*p* < 0.05) by formulation (Table 1) and could be considered between low and normal (15% and 40%) using comparable fresh meat products as the standard [26,27]. Samples with normal fat content (P-NF) exhibited greater (*p* < 0.05) weight loss (Table 1) than the reduced-fat samples, regardless of the type of fat. According to the literature, weight loss from culinary treatments tends to decrease as fat content decreases [28]. A comparison of reduced-fat sausages showed that those formulated with EGs (C-RF and O-RF), regardless of whether they contained chia or oat, lost less weight (up to 33% less than those made only with animal fat) (Table 1). It is important to note that these weight loss values are much lower than the ones observed for other reduced-fat fresh meat products where different structured lipid systems were used as animal fat replacers [26,27]. Moreover, our weight loss results are in accordance with water and fat binding properties associated with the thermal process (70 °C for 30 min in a water bath) in raw longanizas as samples reformulated with chia or oat EGs as fat replacers lost less weight than samples elaborated with all-animal fat [20].

These results show that EGs are suitable fat replacers if the aim is to maintain a high level of fat and water in the final product after standard preparation on an electric grill. This may result in greater juiciness in the samples containing EGs as it has been shown that juiciness and cooking loss are negatively correlated, implying that low cooking loss results in greater juiciness [29]. Moreover, the lower cooking loss found in C-RF and O-RF could imply higher nutrient and bioactive compound stability [21]. This means that the use of EGs would result in greater retention of chia and oat bioactive compounds (α-linolenic acid, β-glucans, insoluble fiber, minerals, etc.) [16,17].

### 3.2. Composition and Energy Content of Longanizas

Longanizas are fresh meat products and must be cooked before eating and this could alter the concentration of some of their components [21]. Therefore, we have evaluated modifications in their composition resulting from grilling on an electric pan.

#### 3.2.1. Proximate Composition

Slight formulation-related differences were observed in the composition of raw samples. Raw normal fat samples (P-NF) had the lowest (*p* < 0.05) moisture content (52%). However, a comparison among reduced-fat samples only (which ranged between 74 and 66%) showed that those prepared with EGs had the lowest (*p* < 0.05) values (approximately 66%). All raw longanizas had similar (*p* > 0.05) ash (~3%) and protein contents (13–14%). However, two different (*p* < 0.05) formulation-related fat levels were observed in raw samples: 30% in normal fat samples and approximately 13% in reduced-fat ones, regardless of whether they were made with animal fat or EG according to experimental design.

More differences were observed in the proximate composition of samples after grilling (Table 1). These significant differences could be mostly attributable to weight loss (Table 1) during the grilling process [21].

Regarding moisture content, cooked samples performed similarly to raw longanizas. In other words, reduced-fat samples exhibited higher (*p* < 0.05) values and, of these, samples with EG had the lowest (*p* < 0.05) moisture, regardless of whether they contained chia or oat (Table 1). These results coincide with observed weight losses (Table 1). Other authors have previously indicated that the moisture content of fresh grilled meat products such as patties, formulated in the same way as longanizas (by replacing animal fat with different types of emulsions), was lower than normal fat samples formulated with all-animal fat [26,27,30].

After cooking, normal fat samples (P-NF) exhibited the highest (*p* < 0.05) ash levels (related to mineral content) (Table 1). Among reduced-fat samples, those with EGs (C-RF and O-RF) had higher (*p* < 0.05) values than those with animal fat (P-RF) (Table 1). This could be because they lose less weight (Table 1) which implies lower mineral loss [31]. It must also be considered that these longanizas contain EGs made with chia and oat, ingredients with a high mineral content [16,17], which could account for the higher ash values observed in C-RF and O-RF (Table 1).

Cooked samples prepared with all-animal fat (P-NF and P-RF) had higher (*p* < 0.05) protein content than C-RF and O-RF, which had similar values regardless of whether they contained chia or oat (Table 1). This protein content in cooked longanizas could be related to the weight losses associated with each type of longaniza (Table 1) which, in turn, was possibly the result of moisture loss (drip and evaporation) and fat melting during cooking. In contrast to raw samples, three fat levels were observed in cooked samples (Table 1): normal fat samples (P-NF) with the highest (*p* < 0.05) fat content, and two significantly different reduced-fat levels, one higher level (*p* < 0.05) corresponding to C-RF and the other with similar values (*p* < 0.05) for P-RF and O-RF (Table 1).

Considering that the samples formulated with EGs (C-RF and O-RF) lost the same amount of weight (Table 1), it is worth noting that EG made with chia has a greater capacity to retain lipid content after grilling. A similar phenomenon has been described by other authors who observed a greater capacity to retain fat as a result of heat treatment in reduced-fat patties made with structured lipid materials as animal fat replacers when compared to samples made with animal fat only [26,27]. It is also interesting to note that, as a result of cooking, lipid content decreased by 60% in normal fat sausages compared to a 16–30% reduction in the case of P-RF, O-RF and C-RF.

#### 3.2.2. Fatty Acid Profile

**Figure 2:**
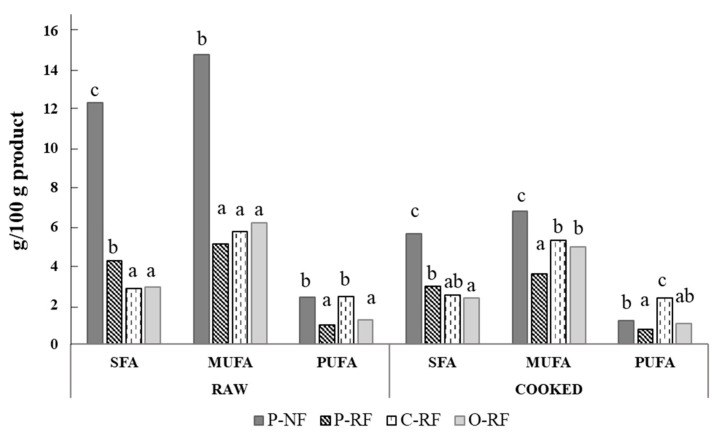
Saturated fatty acid (SFA), monounsaturated fatty acid (MUFA) and polyunsaturated fatty acid (PUFA) (g/100 g of product) of raw and grilled cooked longanizas. For samples denominations, see Figure 1. Different letters (a, b, c), for the same type of fatty acid (SFA, MUFA or PUFA) and treatment of samples (raw or cooked), indicate significant differences (*p* < 0.05) between different formulations (P-NF, P-RF, C-RF and O-RF).

The fatty acid profile of longanizas, based on their saturated fatty acid (SFA), monounsaturated fatty acid (MUFA) and polyunsaturated fatty acid (PUFA) contents, is shown in Figure 2. Among raw samples, those with normal fat content exhibited the highest (*p* < 0.05) SFA and MUFA values (probably due to the higher lipid content). In reduced-fat samples, no significant differences were observed in MUFA content but SFA levels were higher in samples with all-animal fat (P-RF). Raw longanizas prepared with chia EG (C-RF) had similar (*p* > 0.05) PUFA content to samples with normal fat content (Figure 2), despite their lower (*p* < 0.05) lipid content (Table 1).

Similarly, SFA and MUFA contents in cooked samples were higher (*p* < 0.05) in longanizas with normal fat content. However, among reduced-fat samples, those with chia or oat EGs exhibited higher MUFA values than longanizas with all-animal fat. Furthermore, samples with chia EG (C-RF) also showed the highest PUFA level: in most cases, double that of the others (Figure 2). It is worth highlighting their ALA content (1.09 g/100 g of product) even after cooking. Consequently, samples prepared with chia EG would be a good choice based on the daily dietary intake reference for ALA [32].

Use of these EGs in meat product development could be an interesting strategy for obtaining products with a healthy lipid profile, not only due to their vegetable oil content (such as olive oil) and other ingredients with high levels of healthy oil compounds (such as chia seed), but also due to their high capacity to retain that lipid content during cooking (Figure 2), a property not observed in all-animal fat samples (Table 1).

Evaluation of lipid composition is crucial in cooked products because meat fatty acids melt between about 25 and 50 °C, but SFAs melt at higher and PUFAs at lower temperatures. Moreover, changes in fatty acid concentration (mainly decreased PUFAs) can occur during cooking due to their low oxidative stability [21]. As a consequence of the culinary process applied, both SFA and MUFA content decreased in samples prepared with all-animal fat, ~54% in N-PF and ~30% in R-PF, while the decrease in PUFA was lower (49% and 22%, respectively, in these samples). This behavior was similar in longanizas made with oat EG, where SFA and MUFA contents decreased by approximately 19% for both, while PUFAs decreased by 16%. However, longanizas made with chia EG exhibited different reduction values for SFA, MUFA and PUFA (approximately 12%, 8% and 3%, respectively).

#### 3.2.3. Energy Value

Normal fat samples exhibited the highest energy values in both raw and cooked samples. However, as a consequence of culinary treatment, the energy value in these samples (P-NF) decreased by about 40%, from 325 to 195 Kcal/100 g of product. This is due to the decreased fat content as a consequence of cooking. All raw reduced-fat samples (R-RF, C-RF and O-RF) exhibited energy values between 140 and 160 Kcal/100 g product which changed very little after grilling (approximately 130–140 Kcal/100 g of product).

#### 3.2.4. Overall Nutritional Value: Nutrition and Health Claims

**Figure 3:**
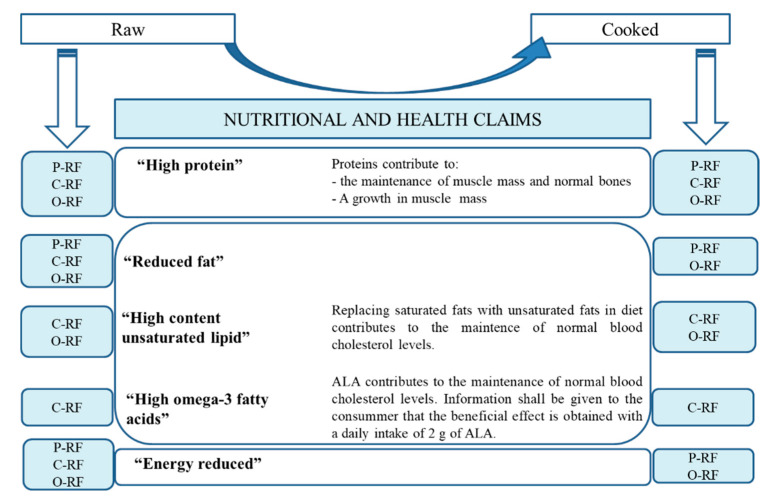
Nutritional and health claims of raw (left) and grilled cooked (right) longanizas. For samples denominations, see Figure 1; ALA: α-linolenic fatty acid.

The initial contact that consumers have with food products is typically through their label. Hence, labels are important in creating consumer expectations. Products whose labels feature nutritional and health claims could improve consumer perception. These claims are made based on product composition at the time of purchase. Therefore, it is important to note that labels on raw reduced-fat longanizas, mainly those made with EGs, are allowed nutritional and health claims (Figure 3) under European legislation [18,19]. However, Article 10 of Regulation (EC) No 1924/2006, which lays down specific conditions for the permitted use of authorized health claims, considers it necessary and important to communicate the way the food is consumed, for example, after being cooked. In other words, consumers must be fully informed with regard to health claims made. Considering that cooking has an impact on the composition of longanizas, it stands to reason that analyses should be performed to ensure that these nutritional and health claims also apply to the cooked product. That is precisely why we have analyzed the impact that grilling has on the nutritional and health claims made with respect to raw longanizas. It is worth noting that this culinary procedure (grilling) had little or no effect on nutritional and health claims (Figure 3). Differences between raw and cooked products were only found in some samples with regard to “reduced fat” and “energy reduced” nutritional claims (Figure 3). Specifically, samples containing chia EG (C-RF) could not be labeled as “reduced fat” or “energy reduced” products after grilling on an electric pan, possibly due to the lower fat loss. Nevertheless, this could be a positive characteristic insofar as these samples maintain high ALA levels which benefit consumers [33].

### 3.3. Technological Properties

#### 3.3.1. pH

pH values were between 6.09 and 6.39 and 6.18 and 6.44 for raw and cooked sausages, respectively (Table 2). Significant differences in pH values were observed resulting from formulation and culinary practices. Formulation-based differences were observed in samples reformulated with chia EG (C-RF) which showed the highest values for both raw and cooked sausages, while the lowest values corresponded to reduced-fat sausages with all-animal fat (P-RF) (Table 2). In most cases, cooking resulted in higher (*p* < 0.05) pH values. Similar behavior was observed in grilled meat products [34].

#### 3.3.2. Color

Color is important due to the impact it has on consumers’ willingness to purchase meat products, with most finding bright red products more appealing. Instrumental color parameter (L*, a* and b*) values of longanizas were affected by the formulation and culinary procedure applied (Table 2). Regarding formulation, both raw and cooked sausages prepared with EG, regardless of whether they contained chia or oat, exhibited higher (*p* < 0.05) b* values. This could be related to the characteristic color of the olive oil used to make the EGs [16,17] which gave them a yellowish green hue due to pigments (mainly chlorophylls and carotenoids). Color likewise depends on the variety and ripeness of the fruit. Oat EGs did not have an impact on the typical red color of products of this sort because their a* values (for both raw and cooked) were significantly similar to those observed in samples made with animal fat (Table 2). However, both raw and cooked longanizas formulated with chia EGs (C-RF) had the lowest a* values which could be attributable to chia’s dark color [8]. No significantly clear formulation-related differences were found for lightness (L*) (Table 2).

Lightness increased in all samples as a result of thermal treatment and redness decreased (*p* < 0.05) in all cases except for C-RF (Table 2). Parameter b* responded in two different ways: all-animal fat samples exhibited lower values after cooking, while the values of those prepared with EGs remained the same (Table 2).

#### 3.3.3. Texture Analysis

**Figure 4:**
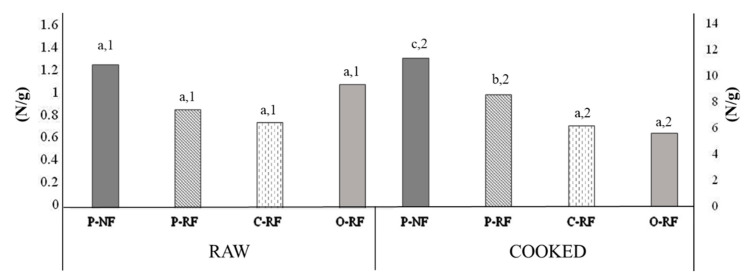
Kramer shear force (KSF, N/g) of raw and grilled cooked longanizas. For samples denominations, see Figure 1. Different letters (a, b, c), for the same treatment of samples (raw or cooked), indicate significant differences (*p* < 0.05) between different formulations (P-NF, P-RF, C-RF and O-RF). Different numbers (1,2) indicate significant differences (*p* < 0.05) between formulations and treatments for each type of sample.

Textural properties of grilled sausages can be useful in the development of a new product because those are the final textural attributes at the moment of consumption. The texture parameters of raw and cooked longanizas are shown in Figure 4. Raw longanizas exhibited similar (*p* < 0.05) KSF values but significant formulation-related differences were observed in cooked samples. KSF values increased for all samples as a result of cooking (Figure 4). Cooked samples made with all-animal fat (P-NF and P-RF) showed higher (*p* < 0.05) KSF values than those made with EGs (Figure 4). This could be related to the fact that normal and reduced-all-animal fat samples showed the highest (*p* < 0.05) weight loss from cooking (Table 1). It has been suggested that higher cooking losses may result in meat products that are more rigid and less easily broken in binding evaluations [35]. Similar textural behavior in terms of increased hardness from cooking was observed by other authors in fresh meat products such as patties reformulated using emulsions and bulking agents as animal fat replacers to improve their fat content [36,37].

#### 3.3.4. Lipid Oxidation Stability

It is well known that thermal treatments such as grilling on an electric pan may accelerate lipid oxidation in meat products, promoting the development of off-flavors and the formation of potentially hazardous compounds. Moreover, high unsaturated fatty acid content, as found in the reformulated sausages used in this study (Figure 2), renders products more susceptible to lipid oxidation [38]. In this context, lipid oxidation levels of raw and grilled longanizas are shown in Table 2. Among raw samples, sausages with all-animal fat showed the highest (*p* < 0.05) lipid oxidation, while no significant differences were observed between reduced-fat samples. Cooking did not affect lipid oxidation in P-NF samples but in reduced-fat samples, it caused lipid oxidation products to accumulate (Table 2). However, the type of fat present in reduced-fat samples affected their oxidation stability and after cooking, those with oat EG (O-RF) showed the highest TBAR values (Table 2). This behavior may be related to the presence of beta-glucan in these samples as it has been shown that the oxidation process of β-glucan is fast at elevated temperatures (85 °C) [39]. Given their composition, lipid oxidation levels in the samples prepared with chia EG (C-RF) were unexpected in comparison with all-animal fat samples. In fact, C-RF even had higher PUFA levels than its all fat counterparts. This could be related to the higher levels of antioxidant compounds in chia seed [40]. Several studies have shown that oxidation caused by thermal treatment is very common in meat products that have been reformulated with lipid materials rich in unsaturated fatty acids [41,42,43].

## 4. Conclusions

Grilling appears to be a suitable cooking method to achieve healthier longanizas that have been formulated using chia or oat EGs as animal fat replacers. Especially after cooking, longanizas prepared with EGs maintained their composition, enabling them to make nutritional and health claims in accordance with European legislation. It should be noted that this reformulation strategy based on chia or oat EGs minimized weight loss during grilling compared with all-animal fat samples, thus improving yield. Furthermore, although the culinary process did modify some technological properties, for the most part, the behavior was similar for samples prepared with all-animal fat and those prepared with EGs.

## Figures and Tables

**Table 1:** Weight losses and proximate composition (%) of grilled cooked longanizas.

Parameters	Samples *			
	P-NF	P-RF	C-RF	O-RF
Weight losses	24.73 ± 0.17 ^c^	22.38 ± 0.27 ^b^	6.71 ± 0.72 ^a^	7.10 ± 1.13 ^a^
Proximate composition				
Ash	3.89 ± 0.08 ^c^	3.27 ± 0.06 ^a^	3.49 ± 0.05 ^b^	3.51 ± 0.03 ^b^
Moisture	62.29 ± 1.04 ^a^	70.93 ± 0.02 ^c^	65.21 ± 0.56 ^b^	66.40 ± 0.20 ^b^
Protein	19.94 ± 0.33 ^c^	17.29 ± 0.34 ^b^	13.61 ± 1.27 ^a^	14.97 ± 0.61 ^a^
Fat	12.76 ± 0.53 ^c^	6.92 ± 0.30 ^a^	9.53 ± 0.75 ^b^	7.92 ± 0.55 ^a^

* For samples denominations, see Figure 1. Means ± standard deviation. Different superscript letters (^a,b,c^) in the same row (for each parameter) indicate significant differences (*p* < 0.05) between different formulations (P-NF, P-RF, C-RF and O-RF).

**Table 2:** pH values, color parameters ((L*) lightness, (a*) redness and (b*) yellowness)) and thiobarbituric acid-reactive substances values (TBARs) (expressed as mg MDA/kg sample) of raw and grilled cooked longanizas.

Parameters	Samples **	Raw	Grilled Cooked
pH	P-NF	6.09 ± 0.06 ^b,1^	6.30 ± 0.02 ^b,2^
	P-RF	6.01 ± 0.00 ^a,1^	6.18 ± 0.01 ^a,2^
	C-RF	6.39 ± 0.01 ^d,1^	6.44 ± 0.02 ^d,1^
	O-RF	6.22 ± 0.01 ^c,1^	6.35 ± 0.02 ^c,2^
Color parameters			
L*	P-NF	49.62 ± 4.82 ^ab,1^	57.21 ± 1.21 ^b,2^
	P-RF	46.22 ± 2.90 ^a,1^	59.21 ± 0.98 ^b,2^
	C-RF	48.52 ± 2.15 ^ab,1^	54.68 ± 0.85 ^a,2^
	O-RF	50.56 ± 1.33 ^b,1^	58.06 ± 1.15 ^b,2^
a*	P-NF	11.46 ± 1.03 ^b,2^	9.47 ± 1.20 ^b,1^
	P-RF	11.32 ± 1.00 ^b,2^	9.78 ± 0.87 ^b,1^
	C-RF	7.11 ± 0.99 ^a,1^	6.30 ± 0.73 ^a,1^
	O-RF	11.40 ± 1.20 ^b,2^	8.72 ± 1.00 ^b,1^
b*	P-NF	6.16 ± 0.80 ^a,2^	4.49 ± 0.61 ^a,1^
	P-RF	5.92 ± 0.43 ^a,2^	4.49 ± 0.38 ^a,1^
	C-RF	8.11 ± 0.94 ^b,1^	7.41 ± 0.39 ^b,1^
	O-RF	8.19 ± 0.70 ^c,1^	7.43 ± 0.97 ^b,1^
TBA(mg MDA/kg sample)	P-NF	0.602 ± 0.08 ^b,1^	0.801 ± 0.151 ^b,1^
	P-RF	0.123 ± 0.04 ^a,1^	0.367 ± 0.093 ^a,2^
	C-RF	0.392 ± 0.03 ^a,1^	0.663 ± 0.140 ^b,2^
	O-RF	0.348 ± 0.08 ^a,1^	2.006 ± 0.241 ^c,2^

** For samples denominations, see Figure 1. Means ± standard deviation. For each parameter, different superscript letters (^a,b,c,d^) in the same column indicate significant differences (*p* < 0.05) between different formulations (P-NF, P-RF, C-RF and O-RF). Different superscript numbers (^1,2^) in the same row indicate significant differences (*p* < 0.05) between formulations and treatments (raw and grilled) for each parameter.
